# Severity in sustained attention impairment and clozapine-resistant schizophrenia: a retrospective study

**DOI:** 10.1186/s12888-019-2204-6

**Published:** 2019-07-12

**Authors:** An-Sheng Lin, Hung-Yu Chan, Ying-Chieh Peng, Wei J. Chen

**Affiliations:** 1 Department of General Psychiatry, Taoyuan Psychiatric Center, Ministry of Health and Welfare, Taoyuan, Taiwan; 2 Office of Superintendent, Taoyuan Psychiatric Center, Ministry of Health and Welfare, Taoyuan, Taiwan; 3 Department of Psychiatry, National Taiwan University Hospital and College of Medicine, National Taiwan University, Taipei, Taiwan; 4 Department of General Psychiatry, Bali Psychiatric Center, Ministry of Health and Welfare, New Taipei City, Taiwan; 5 Institute of Epidemiology and Preventive Medicine, College of Public Health, National Taiwan University, 17 Xu-Zhou Road, Taipei, 100 Taiwan

**Keywords:** Attention, Clozapine, Continuous Performance Test, Schizophrenia, Treatment-resistant

## Abstract

**Background:**

Among patients with treatment-resistant schizophrenia (TRS), some exhibited further clozapine resistance (CR). This study aimed to investigate whether greater severity of treatment resistance in schizophrenia is associated with greater impairments in sustained attention.

**Methods:**

Patients with a DSM-IV-defined schizophrenia were recruited from a psychiatric center in northern Taiwan (April 2010 to October 2010). Both TRS and CR were determined retrospectively from participants’ medical records following the consensus guidelines. The patients were divided into three groups: 102 non-TRS, 48 TRS without CR, and 54 TRS with CR. They underwent both undegraded and degraded Continuous Performance Tests (CPT), and their performance scores (d′) were standardized against a community sample to derive age-, sex-, and education-adjusted z scores.

**Results:**

The TRS with CR group had significantly lower adjusted z scores of d′ on both undegraded and degraded CPTs than the other two groups. Meanwhile, the differences between the TRS without CR group and the non-TRS group were not significant. Multivariable linear regression analyses with adjustment for covariates revealed a trend of gradient impairments on the degraded CPT from non-TRS to TRS without CR and to TRS with CR. The proportions of attentional deficits (an adjusted z score of ≤ − 2.5) on the degraded CPT also exhibited a significant trend, from 36.3% in the non-TRS group to 62.5% in the TRS without CR group and to 83.3% in the TRS with CR group.

**Conclusions:**

Greater severity of treatment resistance in schizophrenia was associated with greater impairments in sustained attention, indicating some common vulnerability.

**Electronic supplementary material:**

The online version of this article (10.1186/s12888-019-2204-6) contains supplementary material, which is available to authorized users.

## Background

Despite extensive progress in the treatment of schizophrenia, approximately 30% of patients of schizophrenia show poor responses to treatment, denoted as treatment-resistant schizophrenia (TRS) [1, 2]. After the initial description of TRS [3], the definition of TRS has evolved over the decades and has converged on well-defined criteria, including response failure to two antipsychotics and persistence of illness despite adequate treatment [1, 4, 5]. Clozapine has remained the gold standard treatment of TRS since its efficacy was described in a randomized trial [6]. However, an estimated 30% of patients receiving clozapine have unsatisfactory responses and are known to have ultra-treatment-resistant schizophrenia [7], super-refractory schizophrenia [8], or clozapine-resistant schizophrenia [5].

Neurodevelopmental pathological processes have been considered to contribute to the etiology of schizophrenia [9–12], and may play a role in the development of TRS [13]. A great deal of research has profiled that TRS has neurochemical and structural abnormalities different from non-TRS or treatment-responsive schizophrenia [8, 14–18]. Hence, TRS might be associated with markers of neurodevelopment. For example, TRS patients have been reported to have certain minor physical anomalies and craniofacial features [19] and more neurological soft signs [20]. Furthermore, part of TRS patients may further encounter clozapine resistance (CR), and it has been suggested that TRS patients with CR might have underlying pathophysiologies that are different from those of TRS patients without CR [16, 17].

A growing body of research indicates that cognitive dysfunction, a core feature of schizophrenia detectable before the onset of full-blown psychosis [21], not only predicts the functional outcomes [22], but also serves as an endophenotype, which is quantitatively measured pathophysiological impairment suggesting genetic susceptibilities to schizophrenia [23]. Among those cognitive dysfunctions, impairments in sustained attention, as measured by the Continuous Performance Test (CPT) [24], are one of the key cognitive domains [25], and also an endophenotype leading to better linkage signals in schizophrenia [26]. CPT impairments are present in patients with schizophrenia across different illness stages [27, 28], not remedied by neuroleptics [29, 30], present in non-psychotic relatives, and have heritability in healthy populations and schizophrenia families [26, 29]. Comparing different cognitive dysfunctions associated with schizophrenia, the magnitude of familial aggregation of CPT impairments is greater than that of executive function impairments measured by Wisconsin Card Sorting Test [26, 31, 32]. Hence, greater CPT impairments may imply more genetic susceptibility to schizophrenia [26], in line with the neurodevelopmental model of schizophrenia [21].

Taken together, aberrant neurodevelopment may underlie both the sustained attention impairments and the development of treatment resistance. Hence, greater attentional impairments might be associated with more severe treatment resistance in schizophrenia. An earlier review found little evidence that patients with TRS had more severe cognitive impairments than patients with non-TRS [33]. However, more recent studies did demonstrate that patients with TRS performed worse on some cognitive domains, e.g., verbal memory [34], attention, cognitive flexibility, processing speed, executive functions, and verbal fluency [35]. Of note, one study comparing TRS patients responsive to clozapine versus TRS patients resistant to clozapine failed to find differences using a battery of neuropsychological tests, including sustained attention, probably due to the small sample sizes and relatively inadequate persistence of the illness [7]. Overall, the literature implies that schizophrenia patients with gradient treatment resistance may have different etio-pathophysiologies, whereas there remains a lack of empirical support from studies that have sufficient sample size and different levels of treatment resistance.

To address the gap in the literature, we sought to examine the severity of sustained attention deficit and the magnitude of treatment resistance in a case-control approach. The aims of the study were to investigate whether greater impairments on the CPT were associated with more treatment resistance in schizophrenia. We conducted two CPT sessions among patients with TRS versus age- and sex-matched patients without TRS and further divided TRS patients into two groups based on the presence of CR or not. We hypothesized that TRS patients with CR would have the greatest CPT impairments, non-TRS patients would have the smallest CPT impairments, and the TRS patients without CR would have moderate CPT impairments.

## Methods

### Participants

This study was part of a larger study investigating TRS conducted in the Bali Psychiatric Center in northern Taiwan from April 2010 to October 2010. The procedure of enrollment has been described in detail in our previous study [19]. Briefly, we examined the medical charts during the study period to recruit patients with schizophrenia according to the DSM-IV criteria and then determined whether the patients had treatment resistance. The study was approved by the Institutional Review Board of the Bali Psychiatric Center (IRB990309–01). We obtained written informed consent from all the participants after providing a complete description of the study. Each subject’s identifier was removed and replaced by a code.

In this study, treatment resistance was defined by the following criteria: 1) a drug-refractory condition; and 2) persistence of illness, which were adapted from the guidelines proposed by Conley and Kelly [4]. Patients who failed to respond to at least two trials of antipsychotics, each for at least 6 weeks and administered at “adequate dosages”, were determined to have a drug-refractory condition. Response failure was determined as worse than or equal to “minimal improvement” in terms of the overall change after switching to a drug on the global improvement subscale of the Clinical Global Impression scale (CGI-I) [36], i.e., CGI-I ≥ 3. The “adequate dosages” were greater than or equal to 600 mg/day of chlorpromazine equivalents (CPZEs) for typical antipsychotics or at adequate dosages based on an expert consensus panel for atypical antipsychotics [37]. Persistence of illness was defined as more than or equal to “moderately ill” on the severity of illness subscale of the Clinical Global Impression scale (CGI-S), i.e., CGI-S ≥ 4. Patients who did not fulfill any of these two criteria were classified as non-TRS, while patients who fulfilled only one criterion were excluded because of grouping controversy.

Moreover, we reappraised the study participants and further divided the TRS patients into two groups based on the presence of CR or not, adapted from the consensus criteria of the Treatment Response and Resistance in Psychosis (TRRIP) working group [5]. Among TRS patients, those who exhibited response failure (i.e., CGI-I ≥ 3) to clozapine at a stable dosage for at least 3 months were thus classified as TRS with CR. Those who had not used clozapine (clozapine never-user) or were rated as better than “minimally improvement” on the CGI-I subscale (i.e., CGI-I < 3) in response to clozapine (clozapine responder) were classified as TRS without CR. Those who had poor responses to clozapine with inadequate durations (i.e., less than 3 months) were excluded from this study.

We further excluded patients if they exhibited the following: 1) a concurrent diagnosis of at least one other major Axis I psychiatric illness (e.g., schizoaffective disorder or substance-induced psychotic disorder); 2) mental retardation; or 3) a parent who was not Han Chinese (e.g., aboriginals or foreigners).

Among the 212 patients included in our previous study (104 non-TRS and 108 TRS) [19], eight patients were excluded, with five due to missing information regarding the CPT and three due to inadequate durations of clozapine trials for grouping. In the final sample of 204 patients, 102 were classified as TRS, who were further divided into TRS without CR (*n* = 48) and TRS with CR (*n* = 54). The remaining 102 patients were classified as non-TRS, who were selected by frequency matching using the distributions of sex and 10-year age groups of the TRS cases.

### Review of medical records

A board-certified psychiatrist (A.-S.L.) performed a systemic review of medical charts to collect clinical information, to rate the CGI scales retrospectively and to determine the group assignments. Considering the medication adherence, only the medication records during hospitalization were used to determine the drug-refractory condition. The group assignment of TRS status for each patient was reconfirmed by the primary care psychiatrist. Only one patient was noted to have TRS assignment incongruent with the determination of the primary care psychiatrist, and was excluded from the study.

### Continuous Performance Test

A computerized version of the CPT that was validated to the procedure of a CPT machine from Sunrise System, version 2.20 (Pembroke, MA, USA), was used to assess sustained attention. The procedure was described in detail elsewhere [38]. In short, numbers from 0 to 9 were randomly presented for 50 milliseconds each, at a rate of one per second. Participants were asked to respond to the target stimulus (the number “9” preceded by the number “1”) by pressing a button. Each participant underwent two CPT sessions: 1) the undegraded 1–9 task; and the 2) 25% degraded 1–9 task. A total of 331 trials, 34 of which were target stimuli, were presented over 5 min for each session. During the 25% degraded session, a pattern of snow was used to make the image less distinct. The sensitivity index of CPT performance (d′) was derived from the hit rate (probability of response to target trials) and the false-alarm rate (probability of response to non-target trials), reflecting an individual’s ability to discriminate target stimuli from non-target stimuli [39, 40].

Furthermore, the d′ was standardized against 345 community controls, with adjustments for age, gender, and education, expressed as adjusted z scores of d′ [41]. We defined a sustained attention deficit by an adjusted z score of less than or equal to − 2.5, as described in a previous family-genetic study [42].

### Statistical analyses

All statistical analyses were performed using the SAS statistical software, version 9.4 (SAS Institute, Cary, NC). For categorical variables, χ^2^ tests or Fisher’s exact tests were used for group comparisons, and a Tukey-type multiple comparison for proportions [43] or multiple comparisons with Fisher’s combination test were used for the relevant post hoc analyses. For continuous variables, t-tests were used in 2 group comparisons, and ANOVA/ANCOVA were used in 3 group comparisons, with Tukey’s HSD tests and Tukey-Kramer adjustments for the post hoc pairwise comparisons. To examine the relations between the adjusted z scores of d′ and the treatment resistance group, which were denoted by dummy variables, we used linear regression analysis with adjustment for covariates.

## Results

The three groups of participants, i.e., non-TRS (*n* = 102), TRS without CR (*n* = 48), and TRS with CR (*n* = 54), were comparable in terms of the distributions of age, sex, educational years, family history of schizophrenia, and current alcohol use status (Table 1). However, there were significant differences among the three groups in terms of the distributions of age of onset, early onset (age of onset younger than 18 years), duration of illness, number of hospitalizations, ratings on the CGI-S, and current cigarette-smoking status. When compared to the non-TRS group, both the TRS without CR group and the TRS with CR group had earlier mean ages of onset, greater proportions of early onset, longer mean durations of illness, greater mean numbers of hospitalizations, and greater mean ratings on the CGI-S. However, there were no significant differences between the two TRS subgroups in terms of these variables.

**Table 1:** Demographic and clinical characteristics of the study participants

	Non-TRS (Group 1)		TRS without CR (Group 2)		TRS with CR (Group 3)		3-Group comparisons^†^	Significant post hoc comparisons^‡^
Variables	(*N* = 102)		(*N* = 48)		(*N* = 54)		p	
	Mean	(SD)	Mean	(SD)	Mean	(SD)		
Age (years)	43.5	(8.6)	45.6	(9.0)	44.6	(8.9)	0.3762	
Age of onset (years)	25.7	(7.4)	22.5	(7.6)	21.6	(5.9)	0.0017	1 > 2, 1 > 3
Education (years)	10.4	(3.2)	9.9	(3.0)	10.4	(2.7)	0.6187	
Illness duration (years)	17.3	(8.8)	23.0	(7.9)	22.9	(8.3)	< 0.0001	1 < 2, 1 < 3
Number of hospitalizations	3.3	(2.9)	4.7	(3.2)	5.2	(3.8)	0.0012	1 < 2, 1 < 3
CGI-S	4.3	(1.3)	5.4	(0.8)	5.7	(0.7)	< 0.0001	1 < 2, 1 < 3
	N	(%)	N	(%)	N	(%)		
Male sex	57	(55.9)	27	(56.3)	30	(55.6)	0.9975	
Early onset (onset age < 18 years)	10	(9.9)	15	(31.3)	15	(27.8)	0.0020	1 < 2, 1 < 3
Family history of schizophrenia	13	(12.8)	6	(12.5)	10	(18.5)	0.5722	
Current habitual cigarette-smoker	49	(48.0)	22	(46.8)	15	(28.3)	0.0496	1 > 3
Current habitual alcohol user	8	(7.8)	0	(0)	1	(1.9)	0.0694	

*TRS* treatment-resistant schizophrenia, *CR* clozapine resistance, *CGI-S* Clinical Global Impression scale-Severity

^†^ANOVA for quantitative variables; χ^2^ test or Fisher’s exact test for categorical variables

^‡^Tukey’s HSD test in ANOVA; a Tukey-type multiple comparison for proportions in a 2*3 cross-tabulation for categorical variables [43]

Table 2 shows that the three groups had remarkably different profiles in terms of residential dispositions and clozapine usage, with the two TRS subgroups having higher proportions of chronic ward inpatients and clozapine usage than the non-TRS group. The mean dosages of clozapine were not significantly different between the two TRS subgroups. The non-TRS group had lower mean antipsychotic dosages of CPZEs and lower proportions of polypharmacy (more than one antipsychotic) and atypical-antipsychotic use (the CPZEs of atypical antipsychotics more than that of conventional antipsychotics) than the two TRS subgroups, while there were no significant differences between the two TRS subgroups.

**Table 2:** Current dispositions and medications of the study participants

	Non-TRS (Group 1)		TRS without CR (Group 2)		TRS with CR (Group 3)		Group comparisons^†^	Significant post hoc comparisons^‡^
Variables	(*N* = 102)		(*N* = 48)		(*N* = 54)		p	
	Mean	(SD)	Mean	(SD)	Mean	(SD)		
Current antipsychotic medication dosage, CPZE, (mg/day)	358.5	(185.8)	458.3	(329.5)	370.8	(186.8)	0.0397	1 < 2
Clozapine current dosage (mg/day)			283.6	(91.9)	322.5	(89.0)	0.0682	
	N	(%)	N	(%)	N	(%)		
Current disposition							< 0.0001	1 ≠ 2 ≠ 3
Chronic ward	9	(8.8)	36	(75.0)	53	(98.1)		
Out-patient department	86	(84.3)	12	(25.0)	1	(1.9)		
Acute ward	7	(6.9)	0	(0)	0	(0)		
Polypharmacy	3	(2.9)	15	(31.3)	12	(22.2)	< 0.0001	1 < 2, 1 < 3
Atypical-antipsychotic user	54	(52.9)	38	(79.2)	50	(92.6)	< 0.0001	1 < 2, 1 < 3
Clozapine use							< 0.0001	1 ≠ 2 ≠ 3
Never-user	102	(100)	17	(35.4)	0	(0)		
Ever-user	0	(0)	2	(4.2)	4	(7.4)		
Current-user	0	(0)	29	(60.4)	50	(92.6)		

*TRS* treatment-resistant schizophrenia, *CR* clozapine resistance, *CPZE* chlorpromazine equivalent

^†^ANOVA for continuous variables in 3-group comparisons; t-test for continuous variables in 2-group comparisons; χ^2^ test or Fisher’s exact test for categorical variables

^‡^Tukey’s HSD test in ANOVA; a Tukey-type multiple comparison for proportions in a 2*3 cross-tabulation for categorical variables [43]; multiple comparisons with Fisher’s combination test in a 3*3 cross-tabulation for categorical variables

Then, the adjusted z scores of CPT d′ among the three groups were compared using ANCOVA with adjustments for illness duration, number of hospitalizations, CGI-S ratings, presence of early onset, smoking status, current antipsychotic dosage, and atypical-antipsychotic use (Table 3). The covariates were selected from the demographic, clinical, and medication variables with group differences, and for covariates that had a pairwise Pearson correlation coefficient of more than or equal to 0.5, we retained the ones with more clinical relevance (Additional file 1: Table S1). We did not adjust for age, sex, or educational years, which were already controlled for in the standardization process; neither did we adjust for current disposition or clozapine usage, which were highly correlated with the determination of treatment resistance status. Regarding the undegraded CPT, the TRS with CR group had significantly lower adjusted z scores of d′ than the other two groups, whereas the TRS without CR group and the non-TRS group exhibited no significant differences for adjusted z scores. Regarding the proportion of sustained attention deficits, it was the highest in the TRS with CR group (81.5%), followed by the TRS without CR group (56.3%), and then the non-TRS group (37.3%). Similarly for the degraded CPT, the TRS with CR group had significantly lower adjusted z scores of d′ than the other two groups, whereas the TRS without CR group and the non-TRS group did not differ significantly in terms of this variable. In terms of the proportion of sustained attention deficits on the degraded CPT, the post hoc comparisons did reach statistical significance, with the highest one in the TRS with CR group (83.3%), followed by that in the TRS without CR group (62.5%), and then that in the non-TRS group (36.3%).

**Table 3:** Adjusted z scores of d′ on the Continuous Performance Test (CPT) among the study participants with group comparisons

	Non-TRS (Group 1)		TRS without CR (Group 2)		TRS with CR (Group 3)		Group comparisons		Significant post hoc comparisons^§^
CPT indices	(*N* = 102)		(*N* = 48)		(*N* = 54)				
Continuous	Mean	(SD)	Mean	(SD)	Mean	(SD)	F	p	
Undegraded CPT									
Adjusted z score of d′	−2.14	(1.98)	−3.31	(2.62)	−4.26	(2.21)	11.91	< 0.0001^†^	1 > 3, 2 > 3
Degraded CPT									
Adjusted z score of d′	−1.73	(1.60)	−2.65	(1.80)	−3.29	(1.19)	10.88	< 0.0001^†^	1 > 3, 2 > 3
Binary	N	(%)	N	(%)	N	(%)	χ^2^	p	
Undegraded CPT deficit									
Adjusted z score of d′ ≤ −2.5	38	(37.3)	27	(56.3)	44	(81.5)	27.96	0.0001^‡^	1 < 3, 2 < 3
Degraded CPT deficit									
Adjusted z score of d′ ≤ −2.5	37	(36.3)	30	(62.5)	45	(83.3)	33.04	< 0.0001^‡^	1 < 2 < 3

*CPT* continuous performance test, *TRS* treatment-resistant schizophrenia, *CR* clozapine resistance, d′, the sensitivity index of performance on the CPT

^†^ANCOVA for continuous variables with covariates including illness duration, number of hospitalizations, the scale of Clinical Global Impression-Severity, early onset or not, smoking status, current antipsychotic dosage, and atypical-antipsychotic user or not

^‡^χ^2^ test for categorical variables

^§^Tukey-Kramer adjustment for ANCOVA; a Tukey-type multiple comparison for proportions in a 2*3 cross-tabulations [43]

To further estimate the relations between CPT performance and the severity of treatment resistance, we conducted multivariable linear regression analyses of the adjusted z scores of d′ for group status, with adjustments for the demographic and clinical features (Table 4). For the undegraded CPT, both the TRS without CR group and the TRS with CR group had lower mean adjusted z scores of d′ than the non-TRS group, with the regression coefficients being − 0.76 and − 2.25, respectively (only the latter statistically significant). Likewise, for the degraded CPT, both the TRS without CR group and the TRS with CR group had lower mean adjusted z scores of d′ than the non-TRS group, with the regression coefficients being − 0.71 and − 1.53, respectively (both statistically significant).

**Table 4:** Linear regression analysis of the adjusted z scores of d′ on the Continuous Performance Test (CPT) in terms of treatment-resistant status with adjustments for covariates

	Undegraded CPT			Degraded CPT		
Variables	Adjusted R^2^ = 0.1581			Adjusted R^2^ = 0.1874		
	β	(95% CI)	p	β	(95% CI)	p
Illness duration, years	−0.04	(−0.08–0.002)	0.0637	− 0.02	(− 0.05–0.005)	0.1104
Number of hospitalizations	0.06	(− 0.05–0.16)	0.2809	0.02	(− 0.05–0.09)	0.5195
CGI-S	0.04	(− 0.27–0.35)	0.7913	0.15	(− 0.07–0.36)	0.1810
Early onset, < 18 years	− 0.36	(− 1.18–0.46)	0.3872	−0.62	(−1.19 – − 0.04)	0.0351
Current cigarette-smoker	−0.31	(− 0.98–0.36)	0.3590	0.31	(− 0.15–0.78)	0.1868
Current antipsychotic dosage	− 0.0005	(− 0.002–0.001)	0.5001	−0.0006	(− 0.0016–0.0005)	0.3079
Atypical-antipsychotic-user	−0.07	(− 0.91–0.77)	0.8695	−0.16	(− 0.74–0.43)	0.5985
Treatment resistance status						
TRS without CR vs. non-TRS	−0.76	(− 1.69–0.18)	0.1120	−0.71	(− 1.36 – − 0.06)	0.0327
TRS with CR vs. non-TRS	−2.25	(− 3.18 – − 1.32)	< 0.0001	− 1.53	(− 2.18 – − 0.88)	< 0.0001

*CPT* continuous performance test, *CGI-S* Clinical Global Impression scale-Severity, *TRS* treatment-resistant schizophrenia, *CR* clozapine resistance, *β* parameter estimate, *CI* confidence interval

Since the TRS without CR group consisted of 17 clozapine never-users and 31 clozapine responders, the two subgroups were further examined. We found that the two subgroups had no significant differences in terms of their demographic and clinical characteristics (Additional file 1: Table S2) or in terms of current disposition and polypharmacy (Additional file 1: Table S3), except for the experience of clozapine usage. Comparing their CPT performances, the clozapine responders had higher scores on the undegraded CPT than the clozapine never-users did, whereas their performances did not differ on the degraded CPT (Additional file 1: Table S4).

## Discussion

This work is one of the few studies that have examined the sustained attention impairments among TRS patients by further grouping them into TRS without CR and TRS with CR. Our results revealed a gradient impairment in sustained attention from non-TRS to TRS without CR and to TRS with CR, regardless of continuous CPT performance scores or the proportion of binary CPT deficits (an adjusted z score of ≤ − 2.5). These findings helped shed light on the relations between sustained attention impairments and the severity of treatment resistance in patients with schizophrenia.

In this study, we demonstrated that the two TRS subgroups were distinct from the non-TRS group, in that they had earlier mean ages of onset, longer durations of illness, and a greater number of hospitalizations, which were compatible with the findings from a previous review [8]. Additionally, two variables were worthy of further discussion. First, compared to the non-TRS group, the TRS with CR group, whom were almost exclusively recruited from chronic wards, had a lower proportion of current habitual tobacco smokers, which was mostly attributed to the tight regulation of tobacco use in chronic wards. Nevertheless, we included smoking status as a covariate to adjust for the potential influences of tobacco smoking on the cognitive performances among patients with schizophrenia [44, 45]. Second, the clozapine dosages were not as high as those observed in other studies [7] possibly because 400 mg/day was the recommended maximum dosage according to the prescription guidelines issued by National Health Insurance Administration in Taiwan.

Our findings revealed that the attentional impairments among TRS patients could be further characterized by the presence of CR, which leads to the most severe impairments. There have been few studies examining attentional impairments within the subgroup of TRS patients. A previous study examining selective attention, as measured by the Stroop test, found TRS patients to have poorer performance than non-TRS patients [35], a finding that was similar to ours, but the study did not further divide its TRS patients according to CR. Another study that did divide their TRS patients further according to CR failed to find any significant differences in a battery of neuropsychological tests covering multiple cognitive domains among three groups of schizophrenia patients, namely, TRS with CR, TRS with clozapine responsiveness, and non-TRS with responsiveness to first-line antipsychotics [7]. Its results were possibly limited by the relatively small sample sizes in each group (15, 20, and 16, respectively) and by a special request from their two groups of TRS patients, which required an adequate response at recruitment either to clozapine monotherapy or combined antipsychotics.

It is noteworthy that we conducted two sessions of the CPT, with the 25% degraded CPT adding a perceptual load to the task and providing one more dimension for detecting schizophrenia vulnerability [46–48]. There have been various versions of CPT for measuring sustained attention. Besides the two versions used in this study, CPT versions with successive identical pairs of 2–4 digits or shapes (CPT-IP), which add working memory loads [47, 49, 50], have been used widely and adopted in a consensus cognitive battery for multi-site clinical trials of cognition treatment in schizophrenia [25]. A review by the Consortium on the Genetics of Schizophrenia summarized that CPTs with high perceptual or working-memory loads successfully detect neurocognitive deficits among biological relatives of schizophrenia patients, while CPTs with low perceptual or working-memory loads fail to detect such deficits [29]. This may explain our finding that the three groups of patients, i.e., non-TRS, TRS without CR, and TRS with CR, showed a clear trend in both continuous CPT performance scores and binary CPT deficit only on the degraded CPT, whereas on the undegraded CPT, the groups of non-TRS and TRS without CR could not be distinguished with statistical significance.

In this study we standardized the CPT performance against a community healthy control sample with adjustment for age, sex, and education to derive adjusted z scores. Using the same community norm, a previous trajectory analysis of repeated CPT measurements during a follow-up period of 4 to 7 years revealed that patients with severe impairments at baseline, i.e., a mean adjusted z score of < − 2.5, had a trajectory pattern of persistent deficit through follow-ups [51]. Intriguingly, attention/vigilance has been considered a separable factor from other cognitive domains [52], a distinct factor from other endophenotypes [53], and hence a useful indicator for illness development, particularly for a disorder with neurodevelopmental dysfunction [21]. Our findings supported the postulation that more severe treatment resistance (i.e., CR) is associated with greater impairments in sustained attention, hence more genetic susceptibility to schizophrenia. A recent genome-wide association study found that clozapine non-responders indeed had higher polygenic risk scores than clozapine responders [54], echoing our results.

In addition, the TRS without CR group tended to have CPT impairments that fell in-between that of the non-TRS group and the TRS with CR group, although the differences between the non-TRS group and the TRS without CR group reached statistical significance only for the binary deficit on the degraded CPT. One explanation is that the degraded CPT, the more difficult version, has been shown to be more sensitive than the undegraded version in showing the non-amenability of sustained attention deficit to short-term antipsychotic treatment [30, 55] and detecting the attention-related genetic susceptibility to schizophrenia [29, 31, 42, 48]. Furthermore, the TRS without CR group included a subgroup of patients who had not yet tried clozapine, who tended to show poorer CPT performances than clozapine responders, though this difference did not reach statistical significance even on the degraded CPT. Given the small sample size of this subgroup of clozapine never-users, future studies are warranted to examine whether clozapine responders are indeed distinguishable from clozapine never-users.

### Implications

Our findings imply that patients with severe deficits in sustained attention may need early intervention with clozapine. Clozapine has remained the treatment of choice for patients with TRS. To initiate clozapine treatment, current practice guidelines recommend a step-by-step course that may be time-consuming. In addition, a variety of side effects may render clinicians hesitant to initiating clozapine. Using the CPT impairment in terms of adjusted z score as an indicator, we may be able to identify schizophrenia patients with attentional deficits of sufficient severity in the early phase of treatment course that warrants initiating clozapine.

### Limitations

This study had several additional limitations. First, sustained attention is only one aspect of the neurocognitive impairments associated with schizophrenia, although it has been widely used and relatively easy to measure. Using a battery covering several key elements of cognitive domains may provide more comprehensive information. Second, this study used the CGI-I to rate symptom changes and the CGI-S to rate severity at recruitment retrospectively. Using a more comprehensive scale, such as the PANSS [56] or the BPRS [57], in a prospective manner may provide better assessments in future studies. Third, we determined the adequate dosages of antipsychotics, including clozapine, based on chart records rather than on blood drug levels. Nevertheless, as suggested in recent guidelines [5], our retrospective assessments were based only on the medication records during hospitalization to assure patients’ adherence to treatment. Fourth, we treated sustained attention deficit as a state-independent endophenotype in this study and only used the CPZEs of current antipsychotic agents as a covariate of ANCOVA in the group comparisons to control for potential confounding by medications. However, some studies found that antipsychotics might attenuate the severity of attentional deficits [29], which was also found in a trajectory analysis that one trajectory class who had moderate CPT impairments of adjusted z score between − 1.0 and − 2.5 did exhibit improvement in CPT performance during the follow-up of 4 to 7 years [51]. These highlight the importance of CPT impairment severity in distinguishing TRS with CR from TRS without CR. Fifth, anticholinergic agents might be associated with memory impairment [58], but information about such treatment was not collected in this study. Finally, we used non-TRS patients rather than treatment-responsive patients as the comparison group. A recent review suggested that using a well-defined treatment-responsive group as the comparison group may offer a better understanding of the origin of treatment resistance [18].

## Conclusions

In summary, our findings suggested that there is a trend of greater impairments in sustained attention associated with greater severity of treatment resistance in schizophrenia, adding support to the postulation that both might share some common vulnerability. In particular, TRS with CR could be viewed as a more homogenous subtype with the greatest impairment in sustained attention, and future research on this subgroup of TRS is warranted to explore the mechanisms underlying treatment resistance in schizophrenia.

## Additional file

Additional file 1: **Table S1.** Pearson correlation coefficients of pairwise variables among demographic, clinical, and medication features. **Table S2.** Demographic and clinical characteristics in the study participants of TRS without CR. **Table S3.** Current dispositions and medications in the study participants of TRS without CR. **Table S4.** Adjusted z scores of d′ on Continuous Performance Test (CPT) in the study participants of TRS without CR. (DOCX 39 kb)

## Data Availability

The datasets generated and analyzed during the current study are not publicly available due to ethical concerns but are available from the corresponding author on reasonable request.

## Abbreviations

CGI-I: Clinical Global Impression scale, the global improvement subscale
CGI-S: Clinical Global Impression scale, the severity of illness subscale
CPT: Continuous Performance Tests
CPT-IP: Identical Paris Continuous Performance Tests
CPZEs: chlorpromazine equivalents
CR: clozapine resistance
TRS: treatment-resistant schizophrenia
