# Supplementary material for: Severity in sustained attention impairment and clozapine-resistant schizophrenia: a retrospective study
Source: BMC Psychiatry. 2019 Jul 12;19:220. doi: 10.1186/s12888-019-2204-6 (PMC6626410; doi:10.1186/s12888-019-2204-6)
Supplement: Supplementary file 1 — Table S1. Pearson correlation coefficients of pairwise variables among demographic, clinical, and medication features. Table S2. Demographic and clinical characteristics in the study participants of TRS without CR. Table S3. Current dispositions and medications in the study participants of TRS without CR. Table S4. Adjusted z scores of d′ on Continuous Performance Test (CPT) in the study participants of TRS without CR. (DOCX 39 kb) [file 12888_2019_2204_MOESM1_ESM.docx]

**Supporting Information**

**Table S1.** Pearson correlation coefficients of pairwise variables among demographic, clinical, and medication features

**Table S2.** Demographic and clinical characteristics in the study participants of TRS without CR

**Table S3.** Current dispositions and medications in the study participants of TRS without CR

**Table S4.** Adjusted z scores of d′ on Continuous Performance Test (CPT) in the study participants of TRS without CR.

**Table S1**. Pearson correlation coefficients of pairwise variables among demographic, clinical, and medication features^†^

| Variable | 1 | 2 | 3 | 4 | 5 | 6 | 7 | 8 | 9 | 10 | 11 | 12 | 13 | 14 | 15 | 16 |
| --- | --- | --- | --- | --- | --- | --- | --- | --- | --- | --- | --- | --- | --- | --- | --- | --- |
| 1. Age | 1 |  |  |  |  |  |  |  |  |  |  |  |  |  |  |  |
| 2. Age at onset | 0.39^***^ | 1 |  |  |  |  |  |  |  |  |  |  |  |  |  |  |
| 3. Educational years | -0.19 | -0.09 | 1 |  |  |  |  |  |  |  |  |  |  |  |  |  |
| 4. Illness duration | **0.65**^***^ | -0.44^***^ | -0.07 | 1 |  |  |  |  |  |  |  |  |  |  |  |  |
| 5. No. hospitalizations | -0.07 | -0.20^**^ | 0.03 | 0.09 | 1 |  |  |  |  |  |  |  |  |  |  |  |
| 6. CGI-S | 0.02 | -0.32^***^ | 0.05 | 0.29^***^ | 0.21^**^ | 1 |  |  |  |  |  |  |  |  |  |  |
| 7. Male sex^‡^ | -0.08 | -0.04 | 0.08 | -0.05 | -0.02 | 0.03 | 1 |  |  |  |  |  |  |  |  |  |
| 8. Early onset^‡^ | -0.28^***^ | **-0.55**^***^ | -0.06 | 0.19^**^ | 0.09 | 0.24^***^ | -0.03 | 1 |  |  |  |  |  |  |  |  |
| 9. Family history^‡^ | -0.05 | -0.18^**^ | 0.03 | 0.09 | 0.16^*^ | 0.10 | 0.02 | 0.19^**^ | 1 |  |  |  |  |  |  |  |
| 10. Cigarette-smoker^‡^ | -0.12 | 0.07 | -0.10 | -0.16^*^ | 0.08 | -0.17 | **0.50**^***^ | -0.08 | -0.10 | 1 |  |  |  |  |  |  |
| 11. Alcohol-user^‡^ | -0.08 | 0.03 | -0.03 | -0.10 | -0.12 | -0.03 | 0.19^**^ | -0.04 | 0.06 | 0.15^*^ | 1 |  |  |  |  |  |
| 12. Antipsychotic Dos. | -0.05 | -0.13 | 0.03 | -0.004 | -0.05 | 0.11 | 0.10 | -0.004 | -0.07 | 0.10 | 0.06 | 1 |  |  |  |  |
| 13. Chronic ward^‡^ | 0.13 | -0.14 | -0.04 | 0.27^***^ | 0.14^*^ | 0.38^***^ | -0.05 | 0.17^*^ | 0.002 | -0.16^*^ | -0.16^*^ | 0.09 | 1 |  |  |  |
| 14. Polypharmacy^‡^ | 0.06 | -0.01 | -0.02 | 0.07 | 0.04 | 0.14^*^ | 0.06 | -0.03 | -0.09 | -0.11 | 0.04 | **0.53**^***^ | 0.24^***^ | 1 |  |  |
| 15. Aty. antipsychotic^‡^ | -0.08 | -0.13 | 0.01 | 0.04 | 0.14^*^ | 0.22^**^ | -0.01 | 0.18^**^ | -0.10 | -0.08 | -0.06 | -0.36^***^ | 0.29^***^ | -0.15^*^ | 1 |  |
| 16. Clozapine user^‡^ | -0.03 | -0.26^***^ | -0.004 | 0.21^**^ | 0.27^***^ | 0.42^***^ | -0.02 | 0.24^***^ | 0.05 | -0.15^*^ | -0.12 | -0.17^*^ | **0.61**^***^ | 0.12 | 0.46^***^ | 1 |

CGI-S, Clinical Global Impression scale-Severity;

^†^the absolute value of correlation coefficient ≥ 0.5 in bold

^‡^binary variable

^*^p < 0.05, ^**^p < 0.01, ^***^p < 0.001

**Table S2.** Demographic and clinical characteristics in the study participants of TRS without CR

| Variables |  | Clozapine never-user (N=17) | |  | Clozapine responder (N=31) | |  | Group comparisons^†^ |
| --- | --- | --- | --- | --- | --- | --- | --- | --- |
|  |  | Mean | (SD) |  | Mean | (SD) |  | p |
| Age (years) |  | 48.4 | (9.8) |  | 44.0 | (8.2) |  | 0.1114 |
| Age of onset (years) |  | 23.8 | (9.6) |  | 21.8 | (6.3) |  | 0.4509 |
| Education (years) |  | 9.5 | (2.2) |  | 10.2 | (3.3) |  | 0.4447 |
| Illness duration (years) |  | 24.5 | (8.0) |  | 22.2 | (7.9) |  | 0.3353 |
| Number of hospitalizations |  | 3.8 | (3.3) |  | 5.3 | (3.1) |  | 0.1149 |
| CGI-S |  | 5.2 | (0.7) |  | 5.5 | (0.9) |  | 0.3179 |
|  |  | n | (%) |  | n | (%) |  | p |
| Male sex |  | 11 | (64.7) |  | 16 | (51.2) |  | 0.3818 |
| Early onset (onset age < 18 years) |  | 5 | (29.4) |  | 10 | (32.3) |  | 0.8388 |
| Family history of schizophrenia |  | 3 | (17.7) |  | 3 | (9.7) |  | 0.6513 |
| Current habitual cigarette-smoker |  | 10 | (62.5) |  | 12 | (38.7) |  | 0.1214 |
| Current habitual alcohol user |  | 0 | (0) |  | 0 | (0) |  | - |

TRS, treatment-resistant schizophrenia; CR, clozapine resistance; CGI-S, Clinical Global Impression scale-Severity.

^†^T-test for quantitative variables; χ^2^ test or Fisher’s exact test for categorical variables.

**Table S3.** Current dispositions and medications in the study participants of TRS without CR

| Variables |  | Clozapine never-user (N=17) | |  | Clozapine responder (N=31) | |  | Group comparisons^†^ |
| --- | --- | --- | --- | --- | --- | --- | --- | --- |
|  |  | Mean | (SD) |  | Mean | (SD) |  | p |
| Current antipsychotic medications dosage, CPZE, (mg/day) |  | 656.3 | (405.7) |  | 349.8 | (218.8) |  | 0.0086 |
| Clozapine current dosage (mg/day) |  | - |  |  | 283.6 | (91.9) |  | - |
|  |  | N | (%) |  | N | (%) |  | p |
| Current disposition |  |  |  |  |  |  |  | 0.1694 |
| Chronic ward |  | 15 | (88.2) |  | 21 | (67.7) |  |  |
| Out-patient department |  | 2 | (11.8) |  | 10 | (32.3) |  |  |
| Acute ward |  | 0 | (0) |  | 0 | (0) |  |  |
| Polypharmacy |  | 6 | (35.3) |  | 9 | (29.0) |  | 0.6544 |
| Atypical-antipsychotic-user |  | 10 | (58.8) |  | 28 | (90.3) |  | 0.0220 |
| Clozapine use |  |  |  |  |  |  |  | < 0.0001 |
| Non-user |  | 17 | (100) |  | 0 | (0) |  |  |
| Ever-user |  | 0 | (0) |  | 2 | (6.4) |  |  |
| Current user |  | 0 | (0) |  | 29 | (93.6) |  |  |

TRS, treatment-resistant schizophrenia; CR, clozapine resistance; CPZE, chlorpromazine equivalent.

^†^T-test for quantitative variables; χ^2^ test or Fisher’s exact test for categorical variables.

.

**Table S4.** Adjusted z scores of d′ on Continuous Performance Test (CPT) in the study participants with TRS without CR

| CPT indicies |  | Clozapine never- user (N=17) | |  | Clozapine responder (N=31) | |  | Group comparisons |
| --- | --- | --- | --- | --- | --- | --- | --- | --- |
| Continuous |  | Mean | (SD) |  | Mean | (SD) |  | p |
| Undegraded CPT |  |  |  |  |  |  |  |  |
| Adjusted z score of d′ |  | -4.57 | (1.70) |  | -2.62 | (2.79) |  | 0.0020^†^ |
| Degraded CPT |  |  |  |  |  |  |  |  |
| Adjusted z score of d′ |  | -3.13 | (1.33) |  | -2.39 | (1.98) |  | 0.0695^†^ |
| Binary |  | N | (%) |  | N | (%) |  | p |
| Undegraded CPT |  |  |  |  |  |  |  |  |
| Adjusted z score of d′ ≤ -2.5 (deficit) |  | 13 | (76.5) |  | 14 | (45.2) |  | 0.0365^‡^ |
| Degraded CPT |  |  |  |  |  |  |  |  |
| Adjusted z score of d′ ≤ -2.5 (deficit) |  | 12 | (70.6) |  | 18 | (58.1) |  | 0.3914^‡^ |

CPT, continuous performance test; TRS, treatment-resistant schizophrenia; CR, clozapine resistance; d′, the sensitivity index of performance on the CPT.

^†^ANCOVA for continuous variables with covariates including illness duration, number of hospitalizations, the scale of Clinical Global Impression-Severity, early onset or not, smoking status, current antipsychotic dosage, and atypical-antipsychotic-user or not.

^‡^χ^2^ test for categorical variables.
